# Comparative Evaluation of Diagnostic and Management Capabilities of Infiniti AI and ChatGPT-4o in Corneal Diseases

**DOI:** 10.7759/cureus.95163

**Published:** 2025-10-22

**Authors:** Abdulaziz Mohammad, Ali Bulbanat, Faisal Aljassar

**Affiliations:** 1 General Surgery, Farwaniya Hospital, Kuwait City, KWT; 2 Ophthalmology, Kuwait Institute for Medical Specializations, Kuwait City, KWT; 3 Ophthalmology, Mohamed Abdulrahman Al Bahar Eye Centre, Ibn Sina Hospital, Kuwait City, KWT

**Keywords:** artificial intelligence, chatgpt, corneal diseases, infiniti ai, ophthalmology

## Abstract

Background: Artificial intelligence (AI), particularly large language models (LLMs), is rapidly transforming medical education and clinical decision support. Ophthalmology, a specialty heavily reliant on pattern recognition, presents a promising domain for LLM integration. While general-purpose models like ChatGPT-4o have demonstrated strong performance in ophthalmic tasks, domain-specific systems such as Infiniti AI, built with a retrieval-augmented generation (RAG) framework, claim advantages by grounding responses in peer-reviewed ophthalmic literature. This study compares ChatGPT-4o (OpenAI, San Francisco, CA, USA) and Infiniti AI (Sinjab Academy, UAE) in corneal disease case scenarios.

Materials and methods: Twenty corneal cases were selected from the University of Iowa EyeRounds database, covering infectious, inflammatory, degenerative, developmental, and systemic associations. ChatGPT-4o, Infiniti AI, and a fellowship-trained cornea specialist independently evaluated each case. Diagnostic and management responses were scored against American Academy of Ophthalmology preferred practice pattern guidelines using a four-point scale (0-3). Statistical comparisons were performed using paired t-tests and Wilcoxon signed-rank tests.

Results: ChatGPT-4o significantly outperformed Infiniti AI across all categories. Diagnostic accuracy was higher for ChatGPT-4o (2.37 ± 0.81) than Infiniti AI (1.13 ± 0.71, p < 0.001, Cohen’s d = 1.35). Management scores were also superior (2.65 ± 0.65 vs 1.98 ± 0.65, p < 0.001, d = 1.37). Overall, ChatGPT-4o achieved a mean total score of 5.00 ± 1.22 compared with 3.10 ± 1.10 for Infiniti AI (p < 0.001, d = 1.75).

Conclusions: ChatGPT-4o demonstrated greater diagnostic and management accuracy than Infiniti AI in corneal disease scenarios, highlighting the current strength of general-purpose LLMs over specialized retrieval-based systems. Nonetheless, both models remain prone to hallucinations and should serve as adjuncts to, rather than replacements for, expert judgment. Further refinement of ophthalmology-specific models is warranted to improve safety and clinical utility.

## Introduction

Artificial intelligence (AI) has emerged as one of the most transformative technologies in medicine, with rapid progress in large language models (LLMs) expanding their potential role in healthcare delivery and medical education [1]. These models, trained on vast amounts of text data, can simulate human-like reasoning and generate clinically relevant responses, making them attractive tools for supporting diagnosis, management, and patient education across medical specialties [2].

Ophthalmology, being a field that heavily relies on pattern recognition and clinical decision-making, is particularly suited for the integration of AI tools [3]. Recent studies have explored the ability of LLMs such as ChatGPT, Bard, and Bing to answer board-style ophthalmology questions, with results showing variable accuracy across subspecialties [1,4]. In glaucoma-related self-assessment questions, ChatGPT-4o demonstrated the highest accuracy, followed closely by Bing, while Bard and ChatGPT-3.5 underperformed [1]. Similar findings have been reported in studies evaluating AI chatbot responses to neuro-ophthalmology and uveitis case scenarios, where GPT-4 outperformed earlier models in providing accurate diagnostic and management guidance [5,6].

Despite these promising outcomes, concerns remain regarding AI-generated errors, lack of contextual judgment, and the potential propagation of misinformation [7]. While AI models may achieve near-expert accuracy in certain controlled tasks, their performance can vary significantly depending on the complexity of the scenario, the specificity of the question, and the quality of their training data [8]. Furthermore, ethical considerations such as bias, patient privacy, and accountability in clinical decision-making must be addressed before widespread adoption [2,3].

Given the growing integration of AI into ophthalmic education and patient care, it is essential to systematically evaluate and compare the diagnostic and management capabilities of current LLMs. This study aims to assess the performance of Infiniti AI (Sinjab Academy, UAE) and ChatGPT-4o (OpenAI, San Francisco, CA, USA) in corneal disease case scenarios, benchmarking their responses against a corneal specialist and established clinical guidelines.

However, despite these advances, LLMs remain prone to hallucinations, where factually incorrect or fabricated information is presented as clinically sound. In ophthalmology, such errors may mislead diagnosis or management decisions if unchecked. Recognizing this limitation underscores the importance of systematically benchmarking LLM outputs against established guidelines and expert judgment [9-11].

Infiniti AI, an ophthalmology-specific model developed by the Sinjab Academy, leverages a retrieval-augmented generation (RAG) framework that grounds its responses in a curated database of over 800,000 peer-reviewed ophthalmic research papers. While this design offers a theoretically strong foundation for domain-specific applications, our study provides a direct comparison with ChatGPT-4o to assess whether such specialization translates into superior performance [12].

## Materials and methods

Case selection

A total of 20 corneal disease cases were selected from the openly available EyeRounds database provided by the Department of Ophthalmology and Visual Sciences at the University of Iowa [12]. The cases encompassed a broad range of conditions, including but not limited to herpes simplex keratitis, Fabry disease, and megalocornea. Each case description contained patient demographics, chief complaint, history of the presenting illness, relevant systemic and ocular history, and examination findings.

Input to large language models

Each case was presented verbatim as input in separate chats with two models: ChatGPT-4o and Infiniti AI. For diagnostic evaluation, the following standardized prompt was used: “Based on the above information, what is the most likely diagnosis? Please also provide a differential diagnosis, if appropriate, including any conditions that should be considered based on the symptoms and findings.” For management evaluation, a new chat was created for each case with the prompt: “Based on the diagnosis, please provide a comprehensive management plan for this patient.”

Comparator (cornea specialist)

The same case scenarios were independently reviewed by a fellowship-trained cornea specialist, who provided diagnostic and management responses to serve as the reference standard.

Grading criteria

The outputs from ChatGPT-4o, Infiniti AI, and the cornea specialist were graded according to the American Academy of Ophthalmology preferred practice pattern guidelines. Both diagnostic and management responses were scored on a 4-point scale (0-3), reflecting their appropriateness and alignment with guideline-based standards: 0 = incorrect or irrelevant response, 1 = partially correct, with major omissions/errors, 2 = mostly correct, with minor omissions/errors, and 3 = fully correct, guideline-concordant response (Table 1).

**Table 1:** Diagnostic and management responses were graded on a four-point scale (0–3) The scale was developed by the authors.

Responses	3 points	2 points	1 point	0 points
Diagnostic responses	The model or specialist provides the exact diagnosis with supporting details that align with the guidelines	The diagnosis is generally correct but lacks some specificity or minor details that are mentioned in the guidelines	The diagnosis is partially correct but misses key elements or provides alternative diagnoses that are less accurate according to the guidelines	The diagnosis is incorrect or completely misaligned with the guidelines
Management responses	The model or specialist provides a comprehensive management plan that fully adheres to the guidelines, including first-line treatments, follow-up recommendations, and potential complications	The management plan is mostly correct but might miss some secondary treatments, less critical details, or considerations that are mentioned in the guidelines	The management plan is somewhat aligned with the guidelines but lacks major components or suggests treatments that are not recommended as first-line	The management plan is incorrect or suggests treatments that are not aligned with the guidelines

Case scenarios evaluated

The following 20 corneal and external disease entities were included (Table 2).

**Table 2:** Twenty corneal and external disease case scenarios used for evaluation

Category	Cases included
Infectious	*Acanthamoeba* keratitis, *Herpes simplex* keratitis, fungal keratitis, infectious crystalline keratopathy
Inflammatory/immune	Atopic keratoconjunctivitis, phlyctenulosis, ocular rosacea, ocular cicatricial pemphigoid, Cogan syndrome, ocular manifestations of Stevens–Johnson syndrome
Degenerative/dystrophic	Fuchs endothelial dystrophy, calcific band keratopathy, acute corneal hydrops, *Cornea verticillata*
Developmental/structural	Peters anomaly, Chandler syndrome, megalocornea
Other/systemic association	Thygeson superficial punctate keratitis, exposure keratopathy (critically ill), Fabry disease

Analysis

A total of 20 corneal case scenarios were independently evaluated by two LLMs: ChatGPT-4o and Infiniti AI. Each model’s responses were scored across diagnostic and management domains using a standardized grading criterion. The performance differences between the two models were analyzed using paired t-tests and Wilcoxon signed-rank tests to ensure robustness. Effect sizes were calculated using Cohen’s d to assess the magnitude of differences. All statistical analyses were conducted using RStudio (Posit Software, Boston, MA, USA), with significance set at p < 0.05.

## Results

ChatGPT-4o and Infiniti AI evaluated a total of 20 corneal case scenarios. ChatGPT-4o achieved significantly higher scores across all categories compared to Infiniti AI. For diagnosis, ChatGPT-4o (2.37 ± 0.81) outperformed Infiniti AI (1.13 ± 0.71), p < 0.001, with a large effect size (Cohen’s d = 1.35). In management, ChatGPT-4o (2.65 ± 0.65) also outperformed Infiniti AI (1.98 ± 0.65), p < 0.001, Cohen’s d = 1.37. When combining diagnostic and management performance, ChatGPT-4o achieved a total mean score of 5.00 ± 1.22, compared to 3.10 ± 1.10 for Infiniti AI, with p < 0.001 and a very large effect size (Cohen’s d = 1.75). Both paired t-tests and Wilcoxon signed-rank tests confirmed the robustness of these differences (all p < 0.001) (Table 3).

**Table 3:** Performance comparison between ChatGPT-4o and Infiniti AI across diagnostic and management SD: standard deviation

Category	ChatGPT-4o (mean ± SD)	Infiniti AI (mean ± SD)	p-value (t-test)	Cohen’s d (effect size)
Diagnosis	2.37 ± 0.81	1.13 ± 0.71	<0.001	1.35 (large)
Management	2.65 ± 0.65	1.98 ± 0.65	<0.001	1.37 (large)
Total	5.00 ± 1.22	3.10 ± 1.10	<0.001	1.75 (very large)

## Discussion

This study provides a comparative evaluation of ChatGPT-4o and Infiniti AI in diagnosing and managing corneal diseases. Our findings demonstrate that ChatGPT-4o significantly outperformed Infiniti AI across both diagnostic and management domains, achieving higher mean scores with very large effect sizes. These results highlight that while domain-specific models such as Infiniti AI offer theoretical advantages through RAG frameworks, general-purpose LLMs like ChatGPT-4o may currently provide superior clinical utility in ophthalmology.

Diagnostic accuracy

The superior diagnostic accuracy of ChatGPT-4o aligns with prior studies reporting high performance of GPT-4 in ophthalmology-related tasks. For example, GPT-4 has been shown to outperform earlier models in ophthalmology board examinations [1,4], neuro-ophthalmology scenarios [5], and complex uveitis case management [6]. In contrast, Infiniti AI, despite being designed specifically for ophthalmology and trained on over 800,000 peer-reviewed papers, yielded lower diagnostic accuracy. This suggests that the breadth and reasoning ability of general LLMs may currently outweigh the benefits of domain specialization, at least in the absence of fine-tuning and optimization for clinical case reasoning.

Management performance

Infiniti AI performed relatively better in management compared to diagnosis, although still below ChatGPT-4o. This may reflect the strength of retrieval-based systems in recalling standardized treatment protocols, but also highlights limitations in contextual integration of case-specific features. ChatGPT-4o’s generative reasoning may provide an advantage in synthesizing individualized management plans that align with guideline-based care [2,3].

Clinical and educational implications

The integration of LLMs into ophthalmology has the potential to enhance medical education, clinical decision support, and patient counseling [2,3,7]. Our findings suggest that ChatGPT-4o may currently serve as a more effective adjunct in these roles compared to Infiniti AI. Nevertheless, Infiniti AI’s domain-specific design represents an important direction for future development. As retrieval-augmented LLMs improve, they may eventually provide both accuracy and explainability, reducing risks of misinformation [13].

From an educational perspective, tools like ChatGPT-4o can support ophthalmology residents by simulating diagnostic reasoning and management planning across a wide range of cases. Similar applications have been demonstrated in ophthalmology board preparation studies, where GPT-4 provided high accuracy in generating guideline-concordant answers [1,4].

Limitations of AI models

Despite promising results, both models remain prone to hallucinations, where factually incorrect or fabricated responses are presented as accurate [9-11]. Such errors carry significant risk in clinical settings, particularly in ophthalmology, where misdiagnosis can result in irreversible visual loss. Prior evaluations have documented hallucination rates across LLMs in healthcare [9,10], underscoring the need for careful benchmarking against expert-reviewed standards.

Study limitations

This study is limited by the use of only 20 corneal case scenarios from a single open-access database. While the cases were diverse, they may not fully represent the spectrum or complexity of real-world clinical encounters. Additionally, the grading system, although guideline-based, involved a single specialist evaluator, which may introduce subjectivity. Expanding to multicenter, multi-rater studies could provide more robust validation.

Strengths and future directions

A strength of this study is its direct comparison of a domain-specific ophthalmology model with a state-of-the-art general-purpose LLM, using real-world clinical cases. Few studies to date have systematically evaluated Infiniti AI, despite its novel RAG-based design [13]. By benchmarking against ChatGPT-4o and an expert specialist, our results provide early evidence-based guidance for integrating such models into ophthalmic practice and education.

Future research should explore strategies to enhance domain-specific models like Infiniti AI, including fine-tuning for clinical reasoning, multimodal integration of imaging data, and real-time updating with new literature [7,8]. Comparative studies across subspecialties (e.g., retina, glaucoma) and prospective trials in clinical workflows will be essential to establish utility and safety.

## Conclusions

ChatGPT-4o outperformed Infiniti AI in diagnostic and management accuracy for corneal disease cases, suggesting that general-purpose LLMs may currently hold greater promise in ophthalmology applications than domain-specific retrieval-based systems. However, both models carry risks of hallucination and should be used as adjuncts rather than replacements for expert judgment. The ongoing refinement of ophthalmology-specific models, alongside rigorous validation, remains crucial for their safe and effective adoption.
